# 90-Day Complication and Readmission Rates for Geriatric Patients With Hip Fracture at Different Time Points From COVID-19 Positivity: A Database Study

**DOI:** 10.5435/JAAOSGlobal-D-24-00069

**Published:** 2024-09-18

**Authors:** Joshua G. Sanchez, Will M. Jiang, Meera M. Dhodapkar, Zachary J. Radford, Lee E. Rubin, Jonathan N. Grauer

**Affiliations:** From the Department of Orthopaedics and Rehabilitation, Yale University, New Haven, CT.

## Abstract

**Introduction:**

Geriatric patients with hip fracture are at risk of having COVID-19 while needing fracture treatment. Understanding the associated risks of variable timing of COVID-19 before surgery may help direct care algorithms.

**Methods:**

Geriatric patients with documented hip fracture surgery were identified within the PearlDiver M157 database. Patients with a preoperative COVID-19 diagnosis were classified based on time from diagnosis to surgery: ≤ 1 week, > 1 to ≤ 4 weeks, > 4 to ≤ 7 weeks, > 7 to ≤ 10 weeks, and > 10 to ≤ 13 weeks. The association of COVID-19 diagnoses with 90-day complications was evaluated.

**Results:**

Overall, 263,771 patients with hip fracture were identified, of which COVID-19 within 13 weeks of surgery was documented for 976. On multivariable analysis, patients with COVID-19 infection within ≤ 1 week preoperatively demonstrated increased rates of minor adverse events (odds ratio (OR) = 1.50), all adverse events (OR = 1.59), sepsis (OR = 1.70), and pneumonia (OR = 2.35) (*P* ≤ 0.0007 for each). For time points greater than 1 week, there were no differences in complication rates.

**Discussion:**

Patients with COVID-19 within 1 week of hip fracture surgery demonstrated greater odds of 90-day complications. Reassuringly, patients with COVID-19 diagnoses more than 1 week preoperatively were not associated with increased odds of any assessed complication.

As of June 2023, SARS-CoV-2 (COVID-19) has caused an estimated number of 768,187,096 cases worldwide and 103,436,829 cases in the United States.^1^ While the effect of COVID-19 on medical care delivery is markedly decreasing, the question of the effect of COVID-19 diagnoses at variable time points before surgeries remains, especially for fractures that require timely surgery such as geriatric hip fractures.

For a notable period during the COVID-19 pandemic, elective surgeries were delayed for moderate lengths of time, when possible, with the hopes of minimizing perioperative adverse outcomes.^2-5^ However, recommendations regarding case delay after COVID-19 diagnosis have been conflicting, with recommended delays at various institutions ranging from 1 to 12 weeks after a full recovery from a confirmed COVID-19 infection.^6-10^

Most centers have considered geriatric hip fracture surgery to be “urgent” and, therefore, necessary, regardless of time from COVID-19 diagnosis, to facilitate mobilization, address fracture pain, and minimize bed rest morbidity.^11^ Compounding this matter, geriatric patients with hip fracture are at an elevated risk of COVID-19. As such, understanding the correlation of a preoperative COVID-19 diagnosis at variable timing before hip fracture surgery is of interest in this vulnerable population that is already associated with notable morbidity and mortality.^12,13^

Previous retrospective studies have demonstrated that patients with a perioperative COVID-19 diagnosis may have an elevated risks of death and pulmonary complications across multiple surgical specialties, with the geriatric population potentially at higher risk.^8,14,15^ Within orthopaedic surgery, a previous retrospective study by Levitt et al^16^ found perioperative COVID-19 diagnoses from 30 days before to 7 days after hip fracture surgery to be associated with a higher 30-day mortality for geriatric patients. However, this study analyzed complications and all-cause mortality in a relatively small size of 185 total COVID-19–positive patients from 46 sites in the United States.^16^ Furthermore, this study did not analyze the effects of variable timing from COVID-19 diagnosis to hip fracture surgery.^16^ In another related study, Pincavitch et al^17^ found increased adverse events for patients undergoing total joint arthroplasty (TJA) only within 2 weeks of a COVID-19 diagnosis.

This study used a national administrative database to evaluate the risk of 90-day postoperative adverse events after hip fracture surgery in geriatric patients with variable timing of COVID-19 diagnosis before surgery. We hypothesized that patients with a more recent COVID-19 diagnosis immediately before hip fracture surgery would have higher odds of certain postoperative complications.

## Methods

### Study Cohort

This study used the 2010-October 2021 PearlDiver (Colorado) M157 administrative database, a large US insurance database that encompasses records from approximately 157 million orthopaedic patients. Using PearlDiver, the data were accessed for research purposes on June 18, 2023. Owing to the anonymized and aggregated qualities of the output data, the authors of this project did not have access to information that could identify individual patients during or after data collection. As such, our institutional review board concluded that research that uses this data set is exempt from review.

Geriatric patients (older than 65 years) with an International Classification of Diseases, 9th Revision or 10th Revision (ICD-9 and ICD-10, respectively), diagnostic code of a hip fracture were identified. Those with closed femoral head/neck, intertrochanteric, and subtrochanteric fractures were included. Of patients with a diagnostic code of a hip fracture, patients who underwent surgical treatment with percutaneous screws, screw and side plate fixation, screw and intramedullary device, hemiarthroplasty, or total hip arthroplasty were selected based on Current Procedural Terminology coding.

Exclusion criteria were defined as open fractures, new diagnosis of neoplasm or non–COVID-19 infection within 90 days before the hip fracture, and patients who were not active in PearlDiver for at least 90 days postoperatively. Patient characteristics extracted from the database included age, sex, and Elixhauser Comorbidity Index (ECI, a marker of overall comorbidity burden).^18,19^

Patients with a documented preoperative, laboratory-confirmed COVID-19 diagnosis (ICD-10 diagnostic code: U07.1) before hip fracture surgery were identified. While patients from 2010 to October 2021 were included to increase the sample size of the COVID-19–negative control group, all COVID-19–positive patients were identified starting from April 2020 (coinciding with the release of the COVID-19 ICD-10 code). These COVID-19–positive patients were categorized based on time from COVID-19 diagnosis to hip fracture surgery as follows: ≤ 1 week, > 1 to ≤ 4 weeks, > 4 to ≤ 7 weeks, > 7 to ≤ 10 weeks, and > 10 to ≤ 13 weeks.

### Postoperative Adverse Events and Readmissions

90-day postoperative complications were found with ICD coding using methods previously described.^20-23^ Characterized in aggregate and individually, evaluated complications included any adverse events (AAEs), severe adverse events, and minor adverse events (MAEs).

Severe adverse events were defined as at least one occurrence of surgical site infection, sepsis, venous thromboembolism ((VTE) either pulmonary embolism or deep vein thrombosis), or cardiac events (myocardial infarction or cardiac arrest). Minor adverse events were defined as at least one event of pneumonia, urinary tract infection, wound dehiscence, transfusion, acute kidney injury, or hematoma. Any adverse events were defined as at least one event of either a severe or MAE.

90-day readmissions were also identified. These were identified based on the “ADMISSIONS” code within the PearlDiver interface as previously described.^22,24^

### Statistical Analysis

Univariable analysis was used to characterize differences between specified study groups. Continuous variables, including age and ECI, were compared using the Student *t*-test or one-way analysis of variance depending on the number of groups being compared. Patient sex and incidence of 90-day complications and readmissions were compared using the Pearson chi-squared test.

Multivariable logistic regression (controlling for age, sex, and ECI) was then used to determine the independent odds ratios (ORs) and 95% confidence intervals (CIs) of each examined adverse event by comparing patients in each time point category with COVID-19–negative patients.

Statistical analysis was conducted using PearlDiver Bellwether software (PearlDiver, Colorado Springs, Colorado) and GraphPad Prism 9.4.1 (GraphPad Software, San Francisco, California). For comparison of patient characteristics, significance was defined as *P* < 0.05. For univariate and multivariate analyses, a Bonferroni correction was applied to account for multiple comparisons (significance defined as *P* ≤ 0.0007).

## Results

### Demographics

In total, 263,771 geriatric patients undergoing hip fracture surgery were identified from 2010 to October 2021. Of these patients, COVID-19 was diagnosed within 13 weeks before surgery for 976 (2.91% of study patients from 2020 to 2021). Those with COVID-19 were, on average, older (78.49 ± 4.96 vs. 75.29 ± 3.81, *P* < 0.0001), were more often male (33.91% vs. 28.62%, *P* = 0.0003), and had higher ECI scores (9.88 ± 4.32 vs. 6.11 ± 3.96, *P* < 0.0001) (Table 1).

**Table 1:** Descriptive Characteristics of Patients Aged 65 Years or Older With and Without a Confirmed COVID-19 Diagnosis Within 13 Weeks Before Hip Fracture Surgery

	COVID-19 (−)	COVID-19 (+) Preoperatively	*P*
*Number of patients*	262,795	976	
*Age* (mean ± SD)	75.29 ± 3.81	78.49 ± 4.96	**<0.0001**
*Sex*			**0.0003**
Female	187,594 (71.38%)	645 (66.08%)	
Male	75,201 (28.62%)	331 (33.91%)	
*ECI* (mean ± SD)	6.11 ± 3.96	9.88 ± 4.32	**<0.0001**

ECI = Elixhauser Comorbidity Index, SD = standard deviation

Bold entries indicate statistically significant.

Patients were then grouped based on time from COVID-19 diagnosis to hip fracture surgery. COVID-19 was diagnosed at ≤ 1 week from surgery for 507 patients, > 1 to ≤ 4 weeks from surgery for 174 patients, > 4 to ≤ 7 weeks from surgery for 103 patients, > 7 to ≤ 10 weeks from surgery for 125 patients, and > 10 to ≤ 13 weeks from surgery for 67 patients (Table 2).

**Table 2:** Descriptive Characteristics of Patients Older Than 65 Years With a Confirmed COVID-19 Diagnosis Before Hip Fracture Surgery

	COVID-19 (+) Preoperatively (n = 976)					*P*
	≤ 1 Week	> 1 to ≤ 4 Weeks	> 4 to ≤ 7 Weeks	> 7 to ≤ 10 Weeks	> 10 to ≤ 13 Weeks	
*No. of patients*	507	174	103	125	67	
*Age* (mean ± SD)	78.47 ± 4.83	78.39 ± 5.14	78.83 ± 4.96	78.57 ± 5.17	78.18 ± 5.10	**<0.0001**
*Sex*						0.9575
Female	336 (66.27%)	118 (67.82%)	67 (65.05%)	82 (65.60%)	42 (62.69%)	
Male	171 (33.73%)	56 (32.18%)	36 (34.95%)	43 (34.40%)	25 (37.31%)	
*ECI* (mean ± SD)	9.56 ± 4.36	9.84 ± 4.19	9.89 ± 4.02	10.80 ± 4.41	10.70 ± 4.35	**< 0.0001**

ECI = Elixhauser Comorbidity Index, SD = standard deviation

COVID-19 (+) patients were categorized based on the preoperative timing of the COVID-19 diagnosis.

Bold entries indicate statistically significant.

### Postoperative Adverse Events and Readmissions

On univariable analysis, there were differences in aggregated AAEs, severe adverse events, MAEs, and individual adverse events (sepsis, cardiac events, pneumonia, urinary tract infection, acute kidney injury, wound dehiscence, and readmissions) between COVID-19–negative and COVID-19–positive patients within 90 days of surgery (*P* < 0.0007 for all) (Table 3).

**Table 3:** Univariable Analyses^a^ of 90-Day Complications and Readmissions of Patients Older Than 65 Years With and Without a Confirmed COVID-19 Diagnosis Before Hip Fracture Surgery

	COVID (−) (n = 262,795)	COVID-19 (+) Preoperatively (n = 976)					*P*
		≤ 1 Week (n = 507)	> 1 to ≤ 4 Weeks (n = 174)	> 4 to ≤ 7 Weeks (n = 103)	> 7 to ≤ 10 Weeks (n = 125)	> 10 to ≤ 13 Weeks (n = 67)	
*Any adverse events*	109,877 (41.81%)	309 (60.95%)	106 (60.92%)	68 (66.02%)	81 (64.80%)	45 (67.16%)	**<0.0001**
*Severe adverse events​*	33,838 (12.88%)	106 (20.91%)	38 (21.84%)	21 (20.39%)	29 (23.20%)	15 (22.39%)	**<0.0001**
Surgical site infection	3177 (1.21%)	12 (2.37%)	< 11	< 11	< 11	< 11	0.0767
Sepsis	10,487 (3.99%)	49 (9.67%)	19 (10.92%)	< 11	11 (8.80%)	< 11	**<0.0001**
Venous thromboembolism	783 (0.30%)	50 (9.86%)	16 (9.20%)	< 11	< 11	< 11	0.0055
Cardiac events	7894 (2.77%)	21 (4.14%)	< 11	< 11	< 11	< 11	**0.0001**
*Minor adverse events*	97,770 (37.20%)	279 (55.03%)	99 (56.90%)	63 (61.17%)	76 (60.80%)	42 (62.69%)	**<0.0001**
Pneumonia	23,399 (8.90%)	118 (23.27%)	34 (19.54%)	17 (16.50%)	21 (16.80%)	16 (23.88%)	**<0.0001**
Urinary tract infection​	53,193 (20.24%)​	127 (25.05%)​	59 (33.91%)	34 (33.01%)​	41 (32.80%)	22 (32.84%)	**<0.0001**
Acute kidney injury	27,067 (10.30%)​	110 (21.70%)	34 (19.54%)	24 (23.30%)	37 (29.60%)	14 (20.90%)	**<0.0001**
Wound dehiscence	1815 (0.69%)	< 11	< 11	< 11	0 (0.00%)	< 11	**<0.0001**
Transfusion	26,059 (9.92%)	41 (8.09%)	18 (10.35%)	11 (10.68%)	12 (9.60%)	13 (19.40%)	0.1187
Hematoma	2499 (0.95%)	< 11	< 11	< 11	< 11	< 11	0.1186
*Readmissions*	43,073 (16.39%)	122 (24.06%)	34 (19.54%)	24 (23.30%)	24 (19.20%)	15 (22.39%)	**<0.0001**

a Bonferroni correction applied, *P* ≤ 0.0007 considered significant.

Bold entries indicate statistically significant.

**Figure 1:**
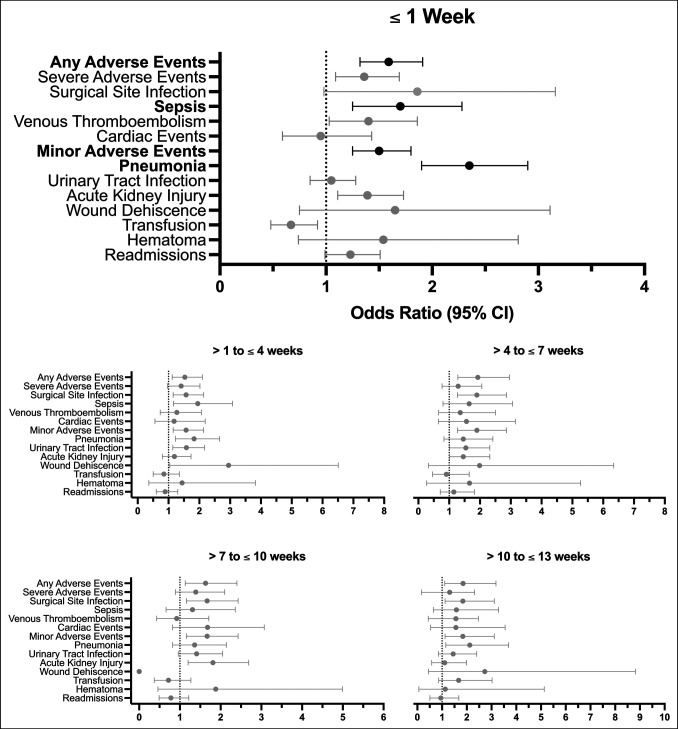
Plots demonstrating risk of 90-day adverse events after hip fracture surgery for geriatric patients with COVID-19 versus without. Forest plots of odds ratios with 95% confidence intervals in the COVID-19–positive cohorts relative to the COVID-19–negative cohort are shown. COVID-19–positive cohorts were categorized based on timing of the COVID-19 diagnosis compared with surgery (as shown in the title of the respective plots). Black bars are statistically significant while gray bars are not. CI = confidence interval.

On multivariable analysis, only patients who underwent hip fracture surgery within 1 week of the COVID-19 diagnosis demonstrated increased odds of 90-day complications compared with COVID-19–negative patients (Table 4, Figure 1). These complications included (in order of decreasing OR) pneumonia (OR 2.35, 95% CI [1.90, 2.90]), sepsis (OR 1.70, 95% CI [1.25, 2.28]), AAEs (OR 1.50, 95% CI [1.32, 1.91]), and MAEs (OR 1.50, 95% CI [1.25, 1.80]) (*P* < 0.0007 for all).

**Table 4:** Multivariable Analyses^a^ (Controlling for Age, Sex, and Elixhauser Comorbidity Index (ECI)) of 90-Day Complications and Readmissions of Patients Older Than 65 Years With a Confirmed COVID-19 Diagnosis Before Hip Fracture Surgery Compared With COVID-19 (−) Patients

	COVID-19 (+) Preoperatively									*P*
	≤ 1 Week OR (95% CI)	*P*	> 1 to ≤ 4 Weeks OR (95% CI)	*P*	> 4 to ≤ 7 Weeks OR (95% CI)	*P*	> 7 to ≤ 10 Weeks OR (95% CI)	*P*	> 10 to ≤ 13 Weeks OR (95% CI)	
*Any adverse events*	**1.59 (1.32, 1.91)**	**<0.0001**	1.53 (1.12, 2.10)	0.0080	1.93 (1.28, 2.96)	0.0020	1.63 (1.13, 2.40)	0.0110	1.85 (1.11, 3.18)	0.0216
*Severe adverse events*	1.36 (1.09, 1.69)	0.0054	1.41 (0.97, 2.02)	0.0633	1.29 (0.77, 2.06)	0.3072	1.39 (0.89, 2.10)	0.1260	1.31 (0.71, 2.31)	0.3577
Surgical site infection	1.86 (0.98, 3.16)	0.0357	0.42 (0.02, 1.87)	0.3844	1.49 (0.24, 4.75)	0.5786	1.69 (0.53, 5.36)	0.3721	2.03 (0.49, 8.40)	0.3272
Sepsis	**1.70 (1.25, 2.28)**	**0.0005**	1.95 (1.16, 3.08)	0.0072	1.65 (0.80, 3.06)	0.1390	1.31 (0.66, 2.36)	0.3959	1.58 (0.65, 3.28)	0.2651
Venous thromboembolism	1.40 (1.03, 1.86)	0.0243	1.27 (0.73, 2.07)	0.3611	1.36 (0.66, 2.50)	0.3564	0.92 (0.43, 1.71)	0.8038	1.56 (0.44, 2.47)	0.7369
Cardiac events	0.95 (0.59, 1.43)	0.8000	1.18 (0.56, 2.19)	0.6300	1.56 (0.65, 3.15)	0.2600	1.68 (0.82, 3.07)	0.1190	1.56 (0.54, 3.55)	0.3450
*Minor adverse events​*	**1.50 (1.25, 1.80)**	**<0.0001**	1.57 (1.15, 2.14)	0.0043	1.90 (1.27, 2.86)	0.0020	1.67 (1.16, 2.43)	0.0067	1.84 (1.11, 3.11)	0.0188
Pneumonia	**2.35 (1.90, 2.90)**	**<0.0001**	1.83 (1.23, 2.66)	0.0019	1.46 (0.83, 2.42)	0.1613	1.36 (0.82, 2.14)	0.2125	2.12 (1.15, 3.69)	0.0108
Urinary tract infection	1.05 (0.85, 1.28)	0.6523	1.58 (1.14, 2.17)	0.0049	1.54 (1.01, 2.32)	0.0420	1.41 (0.96, 2.05)	0.0737	1.45 (0.85, 2.40)	0.1631
Acute kidney injury	1.39 (1.11, 1.73)	0.0036	1.19 (0.80, 1.73)	0.3786	1.46 (0.89, 2.32)	0.1170	1.81 (1.20, 2.69)	0.0038	1.11 (0.58, 1.99)	0.7363
Wound dehiscence	1.65 (0.75, 3.11)	0.1634	2.95 (1.04, 6.51)	0.0178	1.99 (0.33, 6.34)	0.3359	0.00 (0.00, 0.00)	0.8970	2.73 (0.45, 8.82)	0.1641
Transfusion	0.67 (0.48, 0.92)	0.0170	0.85 (0.50, 1.35)	0.5110	0.91 (0.46, 1.65)	0.7810	0.72 (0.37, 1.27)	0.2910	1.67 (0.86, 3.02)	0.1090
Hematoma	1.54 (0.74, 2.81)	0.2010	1.44 (0.36, 3.82)	0.5300	1.66 (0.27, 5.27)	0.4790	1.88 (0.46, 4.99)	0.2840	1.13 (0.06, 5.13)	0.9070
*Readmissions*	1.23 (0.99, 1.51)	0.0554	0.89 (0.60, 1.30)	0.5689	1.15 (0.71, 1.81)	0.5578	0.78 (0.49, 1.22)	0.2954	0.95 (0.51, 1.68)	0.8638

Cohorts were categorized based on timing of the COVID-19 diagnosis.

a Bonferroni correction applied, *P* ≤ 0.0007 considered significant.

Bold entries indicate statistically significant.

No notable differences in 90-day adverse events were observed for patients who received hip fracture surgery after 1 week to the COVID-19 diagnosis (Table 4, Figure 1). 90-day readmission rates were not statistically different for patients in any timing category of COVID-19 diagnosis compared with COVID-19–negative patients.

## Discussion

The correlation of recent COVID-19 diagnosis and perioperative adverse events is not fully defined. Understanding this correlation is very pertinent to geriatric patients with hip fractures for whom surgery is considered necessary and timely.^12,25,26^ Those with COVID-19 within 1 to 12 weeks of surgery have been considered to be at greater risk of perioperative complications.^6,7,9,10,16,17,27^ As such, this study sought to define the complication risks in a large population undergoing hip fracture surgery with varied timing of preoperative COVID-19 infection.

The number of patients with COVID-19 within 13 weeks of hip fracture surgery was relatively small, comprising only 976 cases or 2.91% of surgeries from 2020 to 2021. This low incidence underscores the value of using a large, national database to accrue a population sufficiently powered to address the questions at hand.

Patients with COVID-19 within 13 weeks of hip fracture surgery were older, had higher ECI scores (indicating increased illness severity), and were more likely to be male than those without a COVID-19 diagnosis. These findings align with previous literature that identified older age, male sex, and underlying chronic comorbid conditions as risk factors of COVID-19 in adults.^28,29^

By multivariate analysis, those with a COVID-19 diagnosis within 1 week of hip fracture surgery were at greater odds of pneumonia (OR 2.35) and sepsis (OR 1.70). These individual events contributed to the higher odds of AAEs (OR 1.59) and MAEs (OR 1.50). These findings follow previous research that associated preoperative COVID-19 infection with postoperative pulmonary complications and sepsis after major elective surgery.^27,30,31^ Within orthopaedic surgery, specifically, Pincavitch et al^17^ reported that COVID-19 infection only within 14 days before TJA also increased the risk of pneumonia and sepsis. Furthermore, Galivanche et al^27^ demonstrated that a COVID-19 diagnosis within 2 weeks of hip fracture surgery was associated with increased risk of any, serious, and MAEs. However, this study did not consider the timing of COVID-19 diagnoses and evaluated complications only within 30 days after surgery.^27^ Overall, the findings of this study are consistent with previous literature and suggest that a COVID-19 infection only within 7 days of hip fracture surgery may place patients at higher risk of pneumonia, sepsis, and aggregated adverse events.

There are pertinent negatives for those with COVID-19 diagnosed within 1 week of hip fracture surgery. Critically, this investigation found no notable difference in the rate of VTE, surgical site infection, or cardiac events in any timing category of COVID-19–positive patients compared with COVID-19–negative patients. Regarding VTE, this study parallels the previously mentioned work (Pincavitch et al) that found no difference in the rate of VTE in patients with COVID-19 infection within 90 days of TJA.^17^ However, both these findings contradict previous literature that associates VTE with COVID-19 sequelae.^16,27,32^ With these contrasting findings, additional prospective studies and research are needed to provide robust evidence-based treatment for COVID-19–positive patients with hip fracture.

The lack of complications associated with COVID-19 more than 1 week from hip fracture surgery is a very pertinent finding. Of course, the number and severity of COVID-19 cases have declined after the decreased intensity of the pandemic. Nonetheless, this is thought to only decrease the associated morbidity with surgery in patients with a recent COVID-19 infection and underscores the safety of surgeries done more than 1 week after diagnosis.

This investigation has typical limitations associated with retrospective administrative databases, including concerns regarding data quality and potential unaccounted-for confounding variables. These variables include differences in procedural details, surgeon experience, and the interval between fracture occurrence and surgery. Because data capture of COVID-19 information relied on ICD-10 coding (U07.1), the retrospective design of this study allows for potential error surrounding the diagnosis and time line of COVID-19 and could not account for the severity of COVID-19 diagnoses. In addition, while some of the COVID-19 cases may have been missed based on incorrect administrative coding, these cases would not markedly affect the larger non-COVID-19 patient group. Moreover, this study does not allow for the evaluation of COVID-19 vaccination.

Important to note, the control group of COVID-19–negative patients reflects data from 2010 to October 2021 rather than coinciding with the onset of COVID-19–specific coding in April 2020. This extended time frame was chosen to mitigate the effect of confounding variables by increasing the sample size of COVID-19–negative patients. Furthermore, patients included in this study underwent either fixation or arthroplasty procedures, each associated with different postoperative risks. However, a direct comparison of these surgical methods was not feasible because of insufficient power. Previous research has also demonstrated shifts in hip fracture treatment preferences since 2010, notably the increased use of intramedullary nails over sliding hip screws.^33^ There has also been a general decrease in healthcare utilization factors, such as in-hospital length of stay, mortality, and complication rates after hip fracture surgery since 2010.^33-35^ Finally, although this study uses a large overall population of COVID-19–positive patients, individual study groups remain relatively small. Despite these limitations, this study presents valuable insights into the evolving landscape of geriatric hip fracture treatment beyond 2020, where much of the previous literature ends.

## Conclusion

In conclusion, only patients with a perioperative COVID-19 infection within 1 week of hip fracture surgery demonstrated greater odds of 90-day adverse events and may require specific counseling/care algorithms. Reassuringly, those with COVID-19 diagnosis more than 1 week preoperatively were not associated with increased odds of any of the assessed 90-day adverse events. These data can aid patient counseling and risk stratification for geriatric patients with hip fracture, with potential extrapolation to the surgical treatment of other geriatric injuries. Moreover, these findings highlight the need for additional research to characterize the risks associated with varied timing of COVID-19 diagnoses across different patient populations and procedures.
